# Use and Understanding of Nutrition Labels: Impact of Diet Attachment

**DOI:** 10.3390/foods11131918

**Published:** 2022-06-28

**Authors:** Mar Giró-Candanedo, Anna Claret, Elena Fulladosa, Luis Guerrero

**Affiliations:** IRTA Food Quality and Technology, Finca Camps i Armet s/n, 17121 Monells, Spain; mar.giro@irta.cat (M.G.-C.); anna.claret@irta.cat (A.C.); elena.fulladosa@irta.cat (E.F.)

**Keywords:** dietary habits, food labeling, consumer behavior, nutrition information, Spanish population

## Abstract

Food labels may have an important function in communicating nutrition information and have considerable potential to influence food choice and dietary behavior. Therefore, the aim of this study was to evaluate Spanish consumers’ reasons for reading or not reading nutrition information, their nutrition knowledge, perception and understanding of nutrition label information, and the possible impact of following a diet on all these. A 74-item questionnaire was developed to assess nutrition knowledge, attitude toward food labels, reasons for never reading nutrition information, food choice, the perceived importance of nutrition facts, and label-reading behavior. The results indicated that dietary patterns, nutrition knowledge, and sociodemographic characteristics strongly influenced label use. Based on the participants’ beliefs, four segments were identified for those who followed a diet and three segments for those who did not. Our study suggests that following a diet increases Spanish consumers’ nutrition knowledge as well as their use of nutrition labels, although this cause-effect relationship could be reversed. Nonetheless, further studies would be necessary to clarify the causal direction.

## 1. Introduction

Scientific evidence clearly indicates that our diet and nutrition crucially and substantially affect our health [1]. However, the intake of some food components (e.g., salt, fat, protein, and sugar) can affect each person differently depending on genetic predispositions, age, and diseases [2]. Nutrition labeling has been identified as a promising strategy to help consumers make healthier food choices at the point of purchase and encourage manufacturers to improve the nutrition composition of their products [3]. Nevertheless, the information provided by food labels is sometimes difficult for consumers to understand. Most consumers demand more and better information, although evidence of their ability to adequately understand and interpret such information is insufficient, especially in terms of nutrition composition [4]. Hence, there has been a growing interest in the provision of supplementary, simplified nutrition information in prominent places on the front of packages to help consumers understand the nutritional quality of foods [5]. Under this context, European Union regulation no. 1169/2011 defined food nutrition declaration as mandatory, whereas interpretive front-of-pack nutrition label schemes were determined as an additional voluntary form of providing the consumer with information in an easy-to-use way [6]. To facilitate consumers’ understanding of nutrition information, simpler and more visual front-of-pack labeling emerged in the 1980s in Sweden and Denmark (Nordic Keyhole) and in the 2000s in the Netherlands (Choices) and the United Kingdom (Traffic Lights). New Zealand and Australia, in 2014, introduced the Health Star Rating System, and Chile in 2016 adopted warning symbols for each nutrient whose content is considered too high in food products. Finally, in 2017, French health authorities selected the Nutri-Score as voluntary front-of-pack labeling for pre-packaged foods. However, none of which has been regarded as optimal [7]. Such front-of-pack nutrition labeling is intended to supplement, rather than replace, more detailed nutrition information on the back or side of packs. Generally, as numerous studies have shown, the food industry, government, and public health authorities are keenly interested in knowing and measuring the impact of nutrition claims and associated symbols on individual behavior [8], given the significant economic and welfare implications for society.

Food habits and consumption in Spain have experienced a significant change in recent years. Adherence to a Mediterranean diet has been decreasing among young people in countries with strong Mediterranean traditions, foreshadowing a deteriorating situation in future adults; the situation has also likely been favored by economic factors [9]. One of the most effective ways to reverse this trend may be to increase consumers’ nutrition knowledge of the food products that they typically buy and consume. In this vein, food labels may have an important educational role in making nutritional information accessible to the public. Research consistently suggests an association between healthier diet patterns and the use of nutrition labels. Nutritional claims and symbols are potential aids for the consumer when it comes to identifying which food is the most appropriate option. However, it is unknown how the consumer assimilates and processes that information when purchasing a product and if it affects all individuals and cultures in the same way. Likewise, it is very probable that the same nutritional or health claim will be perceived very differently by different consumers since its perception depends not only on what is indicated or communicated in the claim but also on one’s own beliefs and individual experiences [10]. Being on a diet can positively affect the choice of beneficial nutrient components and negatively affect that of harmful ones [11]. Nevertheless, consumers will generally make better food choices if they understand and use food labels. Therefore, it is important to know the factors in consumers’ use of nutritional information. By identifying these factors, it would be possible to outline the profile of consumers who use or do not use nutritional food labels and nutrient content information, which is a prerequisite for designing food labeling regulations, improving public health, and enhancing the profitability of the food industry [12]. To our knowledge, no study has addressed the possible impact of adherence to dietary patterns on the use of food labels in the Spanish population. Thus, the aim of this study was to evaluate Spanish consumers’ reasons for reading (or not reading) nutrition information, their nutrition knowledge, perception and understanding of the nutrition label information, and the possible impact of dieting on all these.

## 2. Materials and Methods

### 2.1. Participants

A total of 475 consumers were recruited from different Spanish regions through a marketing agency using a filter questionnaire specifically elaborated for this study. Participants had to be responsible or co-responsible for food purchases in their households. Convenience sampling was performed by quotas for sex (minimum 50% women) and age (between 18 and 85 years) attempted to match the Spanish distribution according to the Instituto Nacional de Estadística [13].

### 2.2. Questionnaire

A 74-item questionnaire was developed based on published scales. Participants under 65 years old completed the questionnaire online (*n* = 400), while those aged 65 years or older (*n* = 75) completed the questionnaire using a paper format version. The fieldwork was performed in February 2021. The original questionnaire was written in English and then back-translated into Spanish [14,15].

The questionnaire included eight dimensions of the *Food Choice Questionnaire* (FCQ) [16] (the ethical dimension was not retained since it was considered irrelevant in our case). Each dimension comprised only two items, thus avoiding an excessively long questionnaire. The two selected items within each dimension were selected by consensus between the authors of the present paper, focusing on those items that were more potentially related to the aim of the study. Although an already validated single-item scale for measuring motives for food choice was available [17], we decided to use a simplified version (two items) of the original scale [16] to cover a broader range of the constructs. As stated by Hoeppner et al. [18], when constructs with single items are measured, the respondent faces greater ambiguity in interpreting the meaning of the item. The FCQ items were measured on a seven-point Likert-type agreement scale, following Prescott et al. [19] and Cunha et al. [20]. The participants’ *Attitude Toward Food Labels* [21], *Reasons for Never Reading the Nutrition Information* [22], *Perceived Importance of Nutrition Facts* [23], *Label-Reading Behavior* [21], and *Nutrition Knowledge* [24] were included. *Reasons for Never Reading the Nutrition Information* comprised nine items: eight from the original scale and one additional item developed by consensus between the authors (“*I just pay attention to the price*”). The original scale of measurement was a four-point importance-type scale. However, in our case, we decided to use a seven-point Likert-type agreement scale to gain more discriminant power and to have a more parametric measurement. For *Perceived Importance of Nutrition Facts* and *Label-Reading Behavior*, only 11 and 13 items from the original scales, respectively, were retained. Only those items dealing with specific nutrients were kept, with the sole exception of “trans fat” and “calories from fat”. These two items were not included since we thought that they might be difficult for the target population to properly understand. An additional item was included about the attention given to the nutrition facts label when purchasing food products [21]. For *Perceived Importance of Nutrition Facts*, the measurement scale was extended to a seven-point importance-type scale (the original scale was a three-point importance-type scale). Furthermore, for *Label-Reading Behavior*, a five-point scale was used. Regarding *Attitude Toward Food Labels*, the measurement scale was extended to a seven-point Likert-type agreement scale (the original scale was a five-point agreement-type scale). *Nutrition knowledge* was measured using 20 statements on a three-point scale (true/false/do not know), following Dickson-Spillmann et al. [24]. In addition, the questionnaire contained questions about the participants’ education levels (high, medium, and low), weight, and height. A dedicated question on whether they or someone in their households followed a specific diet was also included to split the sample into two main groups. The different constructs, dimensions, and items used in the questionnaire are shown in Appendix A (Table A1).

### 2.3. Data Analysis

To reduce the number of variables and simplify the data analysis and discussion, the items dealing with the same specific nutrient for *Perceived Importance of Nutrition Facts* and *Label-Reading Behavior* were combined into a new construct, *Impact*. The new items contained in the *Impact* construct were obtained by multiplying items for each specific nutrient for *Perceived Importance of Nutrition Facts* and its corresponding *Label-Reading Behavior*. The first item of *Label-Reading Behavior* (LRB. 1: *When you purchase a food product, do you look at the Nutrition Facts label on the package?*) was not included in the *Impact* construct and was independently analyzed, creating two categories: occasional reading (options: never and rarely) and frequent reading (options: often and always). Body Mass Index (BMI) was calculated for each individual. The participants were then classified into four BMI groups [25]: underweight (BMI < 18.5), normal weight (18.5 ≤ BMI ≤ 24.9), overweight (25.0 ≤ BMI ≤ 29.9), and obese (BMI ≥ 30.0). The overall *Nutrition knowledge* score was obtained by summing all of each participant’s right answers (correct responses were scored as 1, while incorrect responses, do-not-know answers, or blanks were scored as 0). Thus, the total score ranged from 0 (lowest possible *Nutrition knowledge*) to 20 (highest possible *Nutrition knowledge*). The participants were classified into six age groups (18–24, 25–34, 35–44, 45–54, 55–64, and ≥65 years) before the different analyses were performed. Internal consistency was assessed using Cronbach’s α coefficient [26] and was calculated for those items that were supposed to measure the same key construct (*Attitude Toward Food Labels* and the different dimensions of the FCQ). A factorial analysis was then performed to evaluate their unidimensionality. When Cronbach’s α coefficient and the unidimensionality were acceptable (α > 0.60 and factorial loads in the same factor > 0.60), the different items of the same construct or dimension were grouped by computing their mean values (composite variable). According to Ursachi et al. [27], a generally accepted rule is that alpha Cronbach of 0.6–0.7 indicates an acceptable level of reliability. Cronbach’s alpha value of 0.60, although being in the limit, could be considered still acceptable. Internal consistency was unacceptable (0.50 and 0.23, respectively) for the dimensions *Convenience* and *Price* of the FCQ; thus, the individual items were retained for the statistical analysis. For the other scales (*Reasons for Never Reading the Nutrition Information*, *Perceived Importance*, and *Label-Reading Behavior*), the internal consistency and unidimensionality were not verified since these dimensions do not necessarily have to be in the same direction, as observed when dealing with individual beliefs [28].

Analysis of variance (ANOVA, Tukey’s post-hoc test) was performed to determine statistical differences (*p* < 0.05) for each scale (*Attitude Toward Food Labels*, *Reasons for Never Reading the Nutrition Information*, *Impact*, and *Nutrition knowledge*) and the dimensions of the FCQ (*Health*, *Mood*, *Sensory Appeal*, *Natural Content*, *Weight Control and Familiarity Ease*, *Proximity*, *Cheap*, and *Value for Money*) regarding sociodemographic data (age group, sex, and education level groups) and the BMI groups. To identify segments of consumers with similar behavioral patterns, an agglomerative hierarchical cluster analysis was performed (Ward’s method and Euclidean distance) on *Reasons for Never Reading the Nutrition Information*. The number of segments to be retained was selected based on the dendrogram obtained by considering the homogeneity within and among the segments and the principle of parsimony. Discriminant analysis was performed to validate the number of clusters retained by considering the number of individuals who were appropriately classified into their respective clusters (i.e., the confusion matrix). The different clusters obtained were profiled using one-way ANOVA and the K-proportions test (Marascuilo procedure) for continuous and categorical variables, respectively.

The data were analyzed using the XLSTAT statistical software, Version 19.6 (2020) (Addinsoft, France).

## 3. Results

### 3.1. Participants

The sociodemographic characteristics of the participants are presented in Table 1. To study the differences between participants, the sample was divided into two groups: participants that followed any type of diet (including reduced weight; gluten-free, etc.) and those that did not. The first column shows the characteristics of the whole sample (*n* = 475), the second the characteristics of the participants who followed a diet (*n* = 298), and the last column the participants who did not follow a diet (*n* = 175). The overall sample comprised 232 (48.8%) males and 243 (51.2%) females. The most frequent age group was the 35–44 range (35.8%). Most participants had a high level of education (54.9%) and normal weight (50.8%). The participants who followed a diet numbered 298, including 149 males (50%) and 149 females (50%). The highest percentage of this subsample had a high level of education (58.4%) and normal weight (49.3%), while the age range of 35–44 years (38.6%) was the most frequent category. The participants who did not follow a diet numbered 175, including 81 males (46.3%) and 94 females (53.7%). Again, the most frequent categories in this subsample were a high level of education (49.1%), normal weight (53.4%), and age range 35–44 years (30.9%).

### 3.2. Overall Results

The ANOVA results for the pooled data set showed that the percentage of variables in which significant differences (*p* < 0.05) were observed ranged from 19.15% for sex to 73.53% for following or not following a diet (results not shown). As expected, the diet attachment was the most discriminant variable within the different scales assessed. For the variables, age group, education, and BMI, these percentages were 40.43%, 51.06%, and 14.89%, respectively.

The results of the statistical analysis showed significant differences (*p* < 0.05) for 25 out of 34 items/constructs (Table 2), depending on whether participants followed a diet. The participants on a diet had a high level of *Nutrition knowledge*, were more in disagreement with the *Reasons for Never Reading the Nutrition Information*, and showed more interest in the *Impact* of specific nutrients than those who did not follow a diet. Moreover, significant differences were observed in two items of the FCQ (*Natural Content* and *Weight Control*); the participants on a diet scored a higher average value than the other participants. No significant differences (*p* > 0.05) were observed in the *Attitude Toward Food Labels* construct. The results showed that the participants who followed a diet read the nutrition label (68.8%) more frequently than those who did not (56.8%).

In addition, significant differences (*p* < 0.05) were observed for age group and educational level between the participants who followed a diet and those who did not. A significantly higher percentage of respondents over 65 years old, as well as those with a lower education level, affirmed that they did not follow a diet. For the other sociodemographic characteristics, no significant differences were observed.

### 3.3. Participants Who Followed a Diet

The statistical analysis results for the participants who followed a diet are shown in Table 3. Significant differences were detected in 20 of the 34 items/constructs included in the questionnaire. There was a positive effect of education level on *Nutrition knowledge*. Significant differences (*p* = 0.0003) were observed between the participants with a high education level (12.8) and those with low (10.4) and medium (11.2) education levels. Although there were no significant differences (*p* = 0.0670) by sex, females (12.5) tended to have a higher level of *Nutrition knowledge* than males (11.7). *Nutrition knowledge* was not significantly influenced by either age group or BMI. *Attitude Toward Food Labels* was not significantly influenced by any of the selected variables. *Reasons for Never Reading the Nutrition Information* was influenced by education level and BMI. Significant differences (*p* < 0.05) were observed between participants with low and high education levels in three items within this construct. Particularly for participants without advanced education, certain barriers appeared to prevent the use of nutrition information. These barriers included a lack of need due to their familiarity with the food products that they ate regularly, a general disinterest in the information that was provided, and a primary interest in price. In addition, participants who were underweight obtained lower average values than the other participants. For the FCQ construct, significant differences (*p* < 0.05) were observed depending on sex, age group, and education level. Females showed more interest in the *Health* (*p* = 0.002), *Mood* (*p* = 0.011), *Proximity* (*p* = 0.006), *Sensory* (*p* = 0.0.03), *Natural* (*p* = 0.004), and *Value for money p* = 0.026) dimensions. Regarding age group, significant differences between the 35–44 and 45–54 participants were observed for the dimension *Mood* (*p* = 0.001), between >44 and 55–44 for *Sensory* (*p* = 0.001), between 35–44 and 45–54 for *Natural* (*p* = 0.001), between 18–24 and >45 for *Value for money* (*p* = 0.0002), between 35–44 and 45–55 for *Weigh control* (*p* = 0.026), and between 25–34 and 55–64 for *Cheap* (*p* = 0.001). Participants with a medium education level and those who were obese also had higher scores in the *Cheap* dimension (*p* = 0.032). The *Impact* construct was influenced by sex and age group. Females were more affected (obtaining high average values) than males regarding fiber, sugar, and carbohydrates. The youngest participants (18–24) were those with a higher mean value for protein content, whereas consumers in the age group of 45–54 years tended to score higher mean values in all the remaining nutrients. In addition, the results show that females, participants in the age groups of 18–24 years and 55–64 years, and participants with a higher education level read food labels more frequently than the rest of the participants.

### 3.4. Participants Who Did Not Follow a Diet

The statistical analysis results for the participants who did not follow a diet are shown in Table 4. Significant differences were detected in 17 of the 34 items/constructs included in the questionnaire. Again, a positive effect of education level on *Nutrition knowledge* was observed (*p* = 0.004). Participants with a high level of education had a higher level of *Nutrition knowledge* than those with the lowest education level; however, in this case, the differences in the mean values were more important than those obtained for the participants who followed a diet. Although there were no significant differences by age group (*p* = 0.500), the participants in the age range of 18–24 years tended to score a higher level of *Nutrition knowledge* than the rest of the participants. *Attitude Toward Food Labels* was only influenced by sex (*p* = 0.043). Males scored a higher mean value (4.5) than females (4.2). Regarding the *Reasons for Never Reading the Nutrition Information* construct, significant differences were observed, depending on age group (NR.2 *p* = 0.022) and education level (NR.2 *p* = 0.015; NR.3 *p* = 0.0002; NR.4 *p* = 0.004; NR.5 *p* = 0.007; NR.6 *p* < 0.0001).

Generally, the participants with a low education level scored higher mean values than those with a high education level. The results showed that the participants without advanced education faced certain barriers that prevented the use of nutrition information. These barriers included the effort required to read and understand the information and the poor readability resulting from the density of the information and the small font size. On the FCQ scale, significant differences were only observed depending on the age group. The importance of *Familiarity* (*p* = 0.002) seemed to increase with participant age. The *Impact* items were influenced by age group and education level. The participants in the age range of 18–24 years tended to be more interested in sugar (*p* = 0.004) and caloric impact (*p* = 0.019) than those over 55 years old. Generally, the participants with a low education level had less interest in the impact of specific nutrients. In addition, the results showed no significant differences between the participants who read the labels occasionally and those who read them frequently.

### 3.5. Cluster Analysis

#### 3.5.1. Participants Who Followed a Diet

Cluster analysis produced four groups of consumers with different response patterns (see Table 5). Based on the four clusters retained, discriminant analysis allowed a correct classification of 90.27% of the respondents into their respective clusters.

Cluster 1, labeled “*Low knowledge*”, included 86 consumers (28.9%) and comprised individuals with a low level of *Nutrition knowledge*. The individuals in this cluster were not familiar with nutrition information (NR.1). Cluster 2 (*n* = 90), the largest group (30.2%), was labeled “*Confident connoisseur of nutrition information*”. The participants in this cluster had a high level of *Nutrition knowledge*, and the *Reasons for Never Reading the Nutrition Information* were of lower relative importance for them than for the other clusters. Generally, the members in Cluster 2 were concerned about health and mood but did not pay attention to price. In addition, 77.8% of the members in this cluster frequently read the nutrition information on food labels. Cluster 3 (23.5%) shared certain characteristics with Cluster 2. This cluster was labeled “*Unobservant connoisseurs*” because most of the participants were not familiar with nutrition information but had a high level of *Nutrition knowledge*. Finally, Cluster 4 (17.4%) comprised people who mainly considered the purchasing price; hence, they were labeled “*Price-sensitive*”. More than half of the consumers in this cluster (53.8%) frequently read the nutrition information on food labels. The consumers in this cluster were not concerned about health and mood.

#### 3.5.2. Participants Who Did Not Follow a Diet

Cluster analysis produced three groups of consumers with different response patterns (see Table 6). Based on discriminant analysis, the solution for these three clusters allowed a classification of 90.29% of the respondents into the respective clusters.

Cluster 1, labeled “*Disinterested*”, included 57 consumers (32.6%) and comprised individuals with a low level of *Nutrition knowledge*. Generally, the members in this group did not consider value for money as one of the reasons for choosing food. Moreover, they had a low level of interest in the *Impact* of specific nutrients, and only 35.1% of them reported being frequent readers of nutrition information. Cluster 2 (34.3%), the largest, comprising individuals who considered that food labels were difficult to understand (NR.5) and believed that reading nutrition information was time-consuming (NR.2). However, they showed the highest level of interest in food labels (NR.4); hence, they were labeled “*Interested*”. Finally, Cluster 3 (33.1%) was labeled “*Informed*” because its members had the highest level of *Nutrition knowledge* and believed that they understood nutrition information (NR.5). In addition, most of this group’s members (75.9%) frequently read nutrition information.

## 4. Discussion

The sociodemographic results showed that our sample of participants was slightly biased in favor of more highly educated individuals (54.9% vs. the expected 38.6% according to the Ministerio de Educación 2019 [29]) and participants between 25 and 44 years old [13]. These biases could be explained by the individuals’ higher self-confidence and willingness to participate in surveys as their education level increased [30] and the use of an online survey in the case of age bias, although we distributed a written version of the questionnaire for those older than 64 years. Internet penetration in Spain is especially high in the age group 18 to 44 years old (>92%) compared to the older age group (penetration between 85% and 51% for people between 45 and 74 years old) [31]. In addition, our sample of participants was slightly biased in favor of obese individuals (10.4% vs. the expected 16.0% according to INE 2020 [32]). Although Sánchez-Benito et al. [33] observed a progressive abandonment of the Mediterranean diet in the Spanish population, other studies have shown that consumers are increasingly demanding healthy foods and trying to follow a more balanced diet [34]. This could explain the high percentage of individuals in the present study who stated that they or someone in their household followed a diet. These results might indicate that the eating habits of the Spanish population were progressively moving again toward a healthier eating behavior [35]. As expected, an important effect of following or not following a diet was observed for nutrition label use and nutrition knowledge in this study.

Consistent with Barreiro-Hurlé et al. [36] and Campos et al. [11], the results obtained in our study showed that the participants who followed a diet were more capable of understanding nutrition labels and tended to use nutrition labels more. The results confirmed the importance of this health-related behavioral factor in influencing label use [12]. According to Cooke et al. [37], Donga et al. [38], and Ayaz et al. [39], nutrition knowledge is a suitable predictor of nutrition label use, probably because nutrition knowledge can facilitate label use by increasing its perceived benefits and label use efficiency [12]. *Reasons for Never Reading the Nutrition Information* was affected by the participants’ dietary habits. The results may reflect that food label users (the participants who follow a diet) are generally more health conscious. Consistent with our results, Hess et al. [40] showed that health-related variables were the most critical group of predictors of reading nutrition information on food labels. Nevertheless, for most of the participants in our study, it turned out that familiarity with a product limited the reading of food labels. Previous studies indicate that familiarity, as a kind of experience, may increase an individual’s perceived self-confidence regarding the information that they already have [41], thus limiting the willingness for and interest in reading food labels. Most consumers are not used to reading nutrition labels [42], and familiarity with them may indicate that they are aware of their content, but probably without knowing much about their meaning [43]. Health was a determinant of food choice for all the participants, although those on a diet were more interested in the *Impact* of specific nutrients (e.g., sugar and caloric impact). Individuals who follow a diet are typically more attentive to their diet, more concerned about specific nutrients, and more aware of what should be limited (e.g., fat and sugar) [44]. However, some differences were observed depending on the participants’ ages. Consumers over 65 years old most frequently stated that they did not follow any type of diet, probably because these participants were more accustomed to traditional products and already followed healthier eating patterns [45]. However, it is important to point out that traditional foods are not always the healthiest option. Comparing the participants who followed a diet with those who did not, education level had a clear positive effect. Similar to the observations in the present study, Drichoutis et al. [12] and Campos et al. [11] reported that as education level rose, so too did the probability of using food labels, and found a consistent link between the use of nutrition labels and dieting. These could explain the barriers observed in the use of nutrition information by participants with a low level of education. Highly educated participants have greater knowledge, are more aware of the *Impact* that certain foods can have on their health, and have a higher ability to comprehend nutrition food labels [12]. However, as observed in the present study, objective knowledge seems to be more related to personal sensitivity toward this kind of information (when a participant is on a diet) than to education level. Nonetheless, it is important to highlight that being on a diet does not necessarily imply a better or high-quality diet.

The clusters identified in this study enabled us to observe the different profiles of the participants. The results obtained for the participants who followed a diet support the hypothesis that nutrition and label knowledge are associated with label use. Campos et al. [11] reported that the causal nature of this association was likely bidirectional: nutrition labels may promote healthier eating, while individuals with healthier diets are more likely to seek out nutrition labels in the first place. In addition, the participants who followed a diet and considered the price to be important while shopping for food were less likely to use food labels and had a low level of *Nutrition knowledge*, probably because searching for nutrition information and lower prices compete. This result may also indicate that price-sensitive respondents care less about the nutrition quality of the food they buy than non-price-sensitive respondents [12]. The frequency of reading labels seems to be related to difficulties in understanding them. These results are consistent with those of other studies that showed that most respondents thought that it was difficult to understand the information included in food labels [46].

## 5. Conclusions

Dietary patterns, nutrition knowledge, and sociodemographic characteristics strongly influence label use. Our study suggests that following a diet increases Spanish consumers’ nutrition knowledge as well as the use of nutrition labels, although this cause-effect relationship could be reversed. However, the results may also show that participants with greater nutritional knowledge may be more aware of the consequences of the diet on health and may be more likely to follow diets or restrictions. Nonetheless, further studies would be necessary to clarify the causal direction. Reading nutrition information is an important tool in driving people’s food choices, so it could be used successfully in health promotion. Nevertheless, more understandable and legible food labeling may increase the likelihood of using food labels by both those who follow a diet and those who do not.

## 6. Limitations

The present study has three main limitations. The first relates to the use of the selected scales. Since most have not been validated, it is recommended to interpret the results with caution. The second limitation is related to the calculation of the BMI. The data used for weight and height were self-reported data by the participants. The last limitation relates to the sizes of some of the clusters obtained, which are too small to draw robust conclusions [47]. Furthermore, it is noteworthy that the sample of participants had different biases compared to the Spanish population, which was expected from the convenient sampling.

## 7. Ethics Statement

The study was approved by the Ethical Committee of the Institute of Agrifood Research and Technology (IRTA), registration number CCSC 11/2021, in accordance with The Code of Ethics of the World Medical Association (Declaration of Helsinki) for experiments involving humans. Each participant furnished a written informed consent to take part in the study.

## Figures and Tables

**Table 1:** Sociodemographic and other characteristics for the whole sample (A), participants who followed a diet (B), and participants who did not follow a diet (C).

(A) Whole Sample		(B) Participants Who Followed a Diet (62.9%)		(C) Participants Who Did Not Follow a Diet (37.1%)	
Main Characteristics (*n* = 475)	(%)	Main Characteristics (*n* = 298)	(%)	Main Characteristics (*n* = 175)	(%)
Sex		Sex		Sex	
Male	48.8	Male	48.8	Male	48.8
Female	51.2	Female	51.2	Female	51.2
Age group (years)		Age group (years)		Age group (years)	
18–24	4.2	18–24	4.2	18–24	4.2
25–34	20.8	25–34	20.8	25–34	20.8
35–44	35.8	35–44	35.8	35–44	35.8
45–54	17.7	45–54	17.7	45–54	17.7
55–64	8.2	55–64	8.2	55–64	8.2
≥65	13.3	≥65	13.3	≥65	13.3
Education level		Education level		Education level	
Low	11.6	Low	11.6	Low	11.6
Medium	33.5	Medium	33.5	Medium	33.5
High	54.9	High	54.9	High	54.9
Body mass index (BMI)		Body mass index (BMI)		Body mass index (BMI)	
Underweight	3.6	Underweight	3.6	Underweight	3.6
Normal weight	50.8	Normal weight	50.8	Normal weight	50.8
Overweight	35.2	Overweight	35.2	Overweight	35.2
Obesity	10.4	Obesity	10.4	Obesity	10.4

**Table 2:** Mean values and *p*-value for each item or construct of the questionnaire and label reading percentages for the participants who did and did not follow a diet.

Construct	Participants Who Followed a Diet (*n* = 298)	Participants Who Did Not Follow a Diet (*n* = 175)	*p*-Value
NK	12.1	10.9	0.0018
ATFL	4.5	4.4	0.1860
NR.1	3.8	4.3	0.0026
NR.2	2.6	3.2	<0.0001
NR.3	2.4	2.9	0.0027
NR.4	2.4	2.8	0.0061
NR.5	2.7	3.5	<0.0001
NR.6	2.9	3.5	0.0005
NR.7	3.0	3.7	<0.0001
NR.8	3.1	3.4	0.0471
NR.9	2.7	3.1	0.0105
FCQ Health	6.0	5.8	0.0803
FCQ Mood	5.8	5.8	0.9534
FCQ Easy	5.1	5.1	0.8165
FCQ Proximity	5.7	5.6	0.3078
FCQ Sensory appeal	5.8	5.9	0.7227
FCQ Natural content	5.5	5.3	0.0222
FCQ Cheap	3.9	3.8	0.4366
FCQ Value for money	5.7	5.7	0.9926
FCQ Weight control	5.4	4.8	<0.0001
FCQ Familiarity	5.1	5.0	0.2443
Fiber impact	18.8	14.7	<0.0001
Sugar impact	24.4	19.1	<0.0001
Saturated fats impact	23.6	20.1	<0.0001
Salt impact	19.0	14.4	<0.0001
Mineral impact	15.8	12.3	<0.0001
Carbohydrates impact	20.5	16.5	<0.0001
Cholesterol impact	20.3	16.8	0.0002
Total fat impact	23.3	20.0	0.0003
Protein impact	19.7	15.4	<0.0001
Caloric impact	23.6	19.4	<0.0001
Vitamin impact	16.2	12.7	<0.0001
* Occasional reading	4.4%	17.6%	<0.0001
* Frequent reading	68.8%	56.8%	0.0085

NK: *Nutrition knowledge*; ATFL: *Attitude Toward Food Labels*; NR: *Reasons for Never Reading the Nutrition Information*; FCQ: *Food Choice Questionnaire*. * The first item of *Label-Reading Behavior*: occasional reading (options: never and rarely) and frequent reading (options: often and always).

**Table 3:** Mean values for each item or construct of the questionnaire, sociodemographic level, BMI, and label reading percentages for the participants who followed a diet.

	Sex		Age Group (Years)						Education Level			Body Mass Index			
	Female	Male	18–24	25–34	35–44	45–54	55–64	≥65	Low	Medium	High	Underweight	Normal Weight	Overweight	Obese
NK	12.5	11.7	12.7	11.7	12.2	12.7	10.7	11.7	10.4 ^b^	11.2 ^b^	12.8 ^a^	14.7	11.9	11.7	13.4
ATFL	4.5	4.5	4.0	4.5	4.5	4.4	4.7	4.6	4.6	4.5	4.5	4.7	4.6	4.4	4.3
NR.1	3.7	3.9	3.9	3.8	3.7	3.8	3.5	4.6	4.6 ^a^	3.8 ^ab^	3.7 ^b^	2.7	3.6	4.0	4.1
NR.2	2.5	2.6	2.3	2.5	2.6	2.3	2.4	3.5	2.8	2.8	2.4	1.3	2.5	2.7	2.7
NR.3	2.5	2.4	2.1	2.6	2.5	2.1	2.0	3.0	2.9	2.7	2.2	1.3	2.3	2.6	2.6
NR.4	2.3	2.5	2.3	2.5	2.4	2.1	2.3	3.2	3.3 ^a^	2.8 ^ab^	2.0 ^b^	1.3	2.3	2.5	2.5
NR.5	2.7	2.8	2.3	3.0	2.6	2.8	2.6	3.2	3.3	2.9	2.6	1.8	2.6	2.9	3.1
NR.6	2.8	3.0	2.9	2.9	3.0	2.8	2.5	3.2	3.2	3.2	2.7	1.3 ^b^	2.8 ^a^	3.1 ^a^	3.2 ^a^
NR.7	2.9	3.1	2.5	3.0	3.0	2.9	2.9	3.3	3.2	3.2	2.8	1.6 ^b^	2.8 ^ab^	3.2 ^a^	3.3 ^a^
NR.8	3.0	3.2	3.0	3.3	3.0	2.8	3.2	3.5	3.3	3.2	3.0	1.7 ^b^	3.0 ^ab^	3.2 ^a^	3.4 ^a^
NR.9	2.6	2.8	2.7	3.0	2.7	2.2	2.2	3.2	3.2 ^a^	3.0 ^ab^	2.4 ^b^	1.7	2.7	2.8	2.7
FCQ Health	6.2 ^a^	5.8 ^b^	5.7	5.9	5.8	6.4	6.4	6.2	5.7	5.8	6.1	6.7	6.0	5.9	6.1
FCQ Mood	5.9 ^a^	5.6 ^b^	5.7 ^ab^	5.7 ^ab^	5.5 ^b^	6.2 ^a^	6.3 ^a^	6.0 ^ab^	5.8	5.6	5.9	6.5	5.7	5.8	5.8
FCQ Easy	5.2	5.0	5.3	5.2	5.0	5.3	5.3	4.7	4.6 ^b^	5.0 ^ab^	5.3 ^a^	5.6	5.0	5.2	5.2
FCQ Proximity	5.9 ^a^	5.5 ^b^	5.5	5.7	5.6	5.9	6.0	6.1	5.4	5.6	5.8	6.1	5.7	5.8	5.8
FCQ Sensory Appeal	6.0 ^a^	5.6 ^b^	5.3 ^b^	5.7 ^b^	5.7 ^b^	6.0 ^ab^	6.5 ^a^	6.2 ^ab^	5.5	5.8	5.9	6.3	5.8	5.9	5.8
FCQ Natural Content	5.7 ^a^	5.3 ^b^	5.7 ^ab^	5.4 ^ab^	5.2 ^b^	5.8 ^a^	6.2 ^a^	5.8 ^ab^	5.2	5.4	5.6	5.9	5.5	5.5	5.5
FCQ Cheap	4.0	3.9	4.4 ^ab^	4.6 ^a^	3.9 ^ab^	3.5 ^ab^	3.5 ^b^	3.6 ^ab^	3.9 ^ab^	4.3 ^a^	3.8 ^b^	2.3 ^b^	3.9 ^a^	4.1 ^a^	4.2 ^a^
FCQ Value for money	5.9 ^a^	5.6 ^b^	4.9 ^b^	5.8 ^ab^	5.5 ^ab^	6.1 ^a^	6.4 ^a^	6.1 ^a^	5.8	5.7	5.8	5.9	5.7	5.7	6.2
FCQ Weight Control	5.5	5.3	5.2 ^ab^	5.3 ^ab^	5.1 ^b^	5.8 ^a^	5.4 ^ab^	5.6 ^ab^	5.2	5.3	5.4	4.7	5.2	5.5	5.6
FCQ Familiarity	5.2	5.1	4.8	5.2	5.0	5.2	5.5	5.2	5.3	5.0	5.1	5.1	5.0	5.2	5.1
Fiber impact	19.9 ^a^	17.6 ^b^	19.3 ^abc^	17.7 ^bc^	18.0 ^bc^	22.4 ^a^	21.5 ^ab^	13.7 ^c^	15.8	19.3	18.9	23.2	19.0	18.5	17.1
Sugar impact	25.5 ^a^	23.3 ^b^	25.9	25.0	22.7	26.5	27.5	22.0	23.1	23.2	25.3	29.1	24.7	23.3	25.4
Saturated fats impact	23.9	23.2	22.5	24.2	21.8	26.5	27.1	21.1	22.2	23.2	24.0	25.2	24.0	22.7	24.2
Salt impact	19.8	18.2	14.6	18.8	17.9	21.5	22.8	17.6	19.0	19.1	18.9	18.0	19.5	18.5	18.4
Mineral impact	16.1	15.5	11.9 ^ab^	15.8 ^ab^	15.9 ^a^	17.9 ^a^	17.8 ^a^	10.4 ^b^	14.6	16.2	15.7	16.5	16.3	15.9	13.0
Carbohydrates impact	21.6 ^a^	19.3 ^b^	18.7 ^ab^	21.7 ^a^	19.0 ^ab^	23.7 ^a^	23.0 ^a^	15.0 ^b^	17.0	20.8	20.8	19.6	20.9	19.6	21.4
Cholesterol impact	20.8	19.8	14.9 ^b^	20.8 ^ab^	18.2 ^b^	24.6 ^a^	22.9 ^ab^	20.0 ^ab^	20.5	20.7	20.0	21.3	20.6	19.7	20.0
Total fat impact	24.3	22.3	23.9	23.9	21.4	26.6	25.9	20.2	22.6	22.6	23.8	25.0	23.9	22.2	24.1
Protein impact	20.2	19.1	24.7 ^a^	20.5 ^ab^	18.1 ^ab^	21.7 ^ab^	21.0 ^ab^	15.5 ^b^	17.6	20.1	19.7	24.3	19.7	19.1	20.4
Caloric impact	24.6	22.7	24.8 ^ab^	24.4 ^ab^	22.3 ^ab^	26.3 ^a^	26.0 ^ab^	18.5 ^b^	22.1	23.8	23.8	25.0	23.7	22.8	25.9
Vitamin impact	15.9	16.5	4.0	4.5	4.5	4.4	4.7	4.6	14.1	17.5	15.8	17.9	16.1	16.9	13.4
* Occasional reading	4.0%	4.7%	0%	2.9%	3.5%	5.5%	0%	18.2%	20.0%	6.1%	1.1%	0% ^a^	2.7% ^ab^	7.2% ^b^	3.3% ^ab^
* Frequent reading	70.5%	67.1%	86.7%	73.9%	60.9%	72.7%	86.4%	54.5%	56.0%	67.7%	71.3%	88.9%	72.6%	60.4	76.7%

^a–c^: Different letters in the same row and sociodemographic variable (sex, age group, and education level) or BMI indicate statistically significant differences (*p* < 0.05). NK: *Nutrition knowledge*; ATFL: *Attitude Toward Food Labels*; NR: *Reasons for Never Reading the Nutrition Information*; FCQ: *Food Choice Questionnaire*. * The first item of *Label-Reading Behavior*: occasional reading (options: never and rarely) and frequent reading (options: often and always).

**Table 4:** Mean values for each item or construct of the questionnaire, sociodemographic level, BMI, and label reading percentages for the participants who did not follow a diet.

	Sex		Age Group (Years)						Education Level			Body Mass Index			
	Female	Male	18–24	25–34	35–44	45–54	55–64	≥65	Low	Medium	High	Underweight	Normal Weight	Overweight	Obese
NK	10.9	10.8	12.4	11.4	11.4	10.7	10.1	10.0	8.8 ^b^	10.5 ^ab^	11.8 ^a^	13.0	10.6	11.0	10.9
ATFL	4.2 ^b^	4.5 ^a^	3.9	4.3	4.3	4.4	4.3	4.6	4.3	4.6	4.3	3.9	4.4	4.5	4.3
NR.1	4.4	4.1	4.6 ^ab^	4.1 ^ab^	4.0 ^b^	4.1 ^ab^	4.0 ^ab^	5.0 ^a^	4.8	4.0	4.2	4.0	4.0	4.6	4.5
NR.2	3.1	3.3	3.2	3.0	2.9	3.1	3.1	3.9	3.8 ^a^	3.4 ^ab^	2.9 ^b^	3.8	3.0	3.3	3.7
NR.3	2.9	2.9	2.0	2.8	2.6	2.7	2.5	3.5	3.8 ^a^	3.0 ^ab^	2.5 ^b^	2.6	2.7	3.0	3.4
NR.4	2.9	2.8	2.6	2.6	2.7	2.8	3.4	3.0	3.7 ^a^	2.8 ^b^	2.5 ^b^	3.8	2.7	2.7	3.4
NR.5	3.4	3.6	3.4	3.6	3.0	3.6	3.7	3.9	4.3 ^a^	3.5 ^ab^	3.2 ^b^	3.6	3.4	3.6	3.5
NR.6	3.4	3.6	3.4	3.3	3.3	3.4	3.4	3.9	4.7 ^a^	3.5 ^b^	3.0 ^b^	3.4	3.3	3.5	4.2
NR.7	3.5	3.9	3.2	3.4	3.5	3.8	3.7	4.1	4.2	3.7	3.5	3.4	3.5	4.0	4.0
NR.8	3.6	3.2	3.6	3.1	3.4	3.3	3.6	3.5	3.9	3.3	3.3	4.3	3.4	3.3	3.5
NR.9	3.1	3.1	2.2	3.3	2.8	3.2	3.3	3.2	3.8 ^a^	3.0 ^ab^	2.9 ^b^	3.3	2.9	3.1	3.7
FCQ Health	5.8	5.7	5.9	5.4	5.8	5.8	5.7	6.1	5.6	5.7	5.9	5.9	5.8	5.8	5.6
FCQ Mood	5.9	5.6	5.5	5.6	5.8	5.5	5.7	6.2	5.8	5.7	5.8	5.1	5.8	5.9	5.6
FCQ Easy	5.3	4.9	5.0	5.5	5.0	4.9	4.8	5.3	5.0	4.8	5.3	5.0	5.1	5.1	4.8
FCQ Proximity	5.6	5.6	6.2	5.4	5.3	5.7	5.6	6.0	5.6	5.5	5.7	5.0	5.5	5.7	5.9
FCQ Sensory appeal	5.9	5.8	5.9	5.6	5.7	5.8	6.0	6.2	5.8	5.8	5.9	5.6	5.9	5.9	5.8
FCQ Natural content	5.4	5.1	5.6 ^ab^	5.0 ^ab^	5.4 ^ab^	5.0 ^ab^	4.5 ^b^	5.7 ^a^	5.1	5.2	5.4	5.0	5.4	5.2	4.9
FCQ Cheap	3.8	3.9	3.2	4.0	3.4	3.8	4.0	4.3	4.1	4.0	3.6	3.1	3.8	3.8	4.2
FCQ Value for money	5.8	5.7	5.8	5.5	5.6	5.8	5.6	6.1	5.7	5.8	5.7	5.6	5.7	5.9	5.6
FCQ Weight control	4.9	4.6	4.3	4.8	4.5	4.9	4.3	5.3	4.8	4.7	4.8	3.8	4.8	4.9	4.8
FCQ Familiarity	5.0	5.0	3.9 ^b^	4.9 ^ab^	4.9 ^ab^	4.7 ^ab^	5.0 ^a^	5.5 ^a^	5.2	5.0	4.9	4.4	5.0	5.1	4.9
Fiber impact	14.6	14.7	16.8	15.4	14.4	16.8	13.2	13.3	11.3 ^b^	16.3 ^a^	14.7 ^ab^	11.6	16.0	14.1	11.7
Sugar impact	20.0	17.9	24.6 ^a^	22.5 ^ab^	19.8 ^ab^	21.3 ^ab^	15.0 ^b^	15.0 ^b^	15.1 ^b^	18.1 ^ab^	21.1 ^a^	21.4	20.9	17.4	14.3
Saturated fats impact	20.6	19.4	23.8	21.0	20.8	21.6	17.2	18.0	17.1 ^b^	19.0 ^ab^	21.8 ^a^	19.5 ^ab^	21.8 ^a^	19.3 ^ab^	14.5 ^b^
Salt impact	14.4	14.3	16.0	14.3	14.3	15.3	11.5	14.8	14.6	14.1	14.5	10.5	15.4	14.3	11.9
Mineral impact	12.1	12.5	15.7	12.8	12.7	14.4	11.9	9.5	9.4 ^b^	13.9 ^a^	12.2 ^ab^	10.6	13.0	12.5	9.3
Carbohydrates impact	16.7	16.4	20.6	17.0	17.2	19.2	14.1	13.9	12.3 ^b^	16.6 ^ab^	18.0 ^a^	14.9	17.8	16.3	12.5
Cholesterol impact	16.5	17.2	16.6	15.4	16.6	18.8	12.5	18.6	16.6	16.4	17.2	11.6	18.0	16.9	13.8
Total fat impact	20.4	19.6	22.0	22.2	20.8	20.6	15.2	18.8	17.7	19.4	21.3	18.0 ^ab^	22.0 ^a^	18.8 ^ab^	14.7 ^b^
Protein impact	15.6	15.3	16.8	17.5	14.9	17.1	11.6	14.9	13.5	16.1	15.7	10.8	17.0	14.9	12.2
Caloric impact	20.1	18.5	23.6 ^a^	20.5 ^ab^	20.9 ^ab^	21.5 ^ab^	13.2 ^b^	16.9 ^ab^	15.7	19.5	20.5	18.5	20.7	18.7	15.8
Vitamin impact	12.7	12.8	17.5	12.4	12.7	14.6	11.4	11.7	12.0	13.7	12.3	11.3	13.3	12.6	11.8
* Occasional reading	20.2%	14.8%	0%	16.7%	20.4%	20.7%	29.4%	10.0%	23.3%	20.3%	14.0%	37.5%	13.8%	16.7%	26.3%
* Frequent reading	58.5%	55.6%	80.0%	53.3%	53.7%	51.7%	47.1%	70.0%	56.7%	49.2%	62.8%	50.0%	61.7%	55.6%	42.1%

^a–c^: Different letters in the same row and sociodemographic variable (sex, age group, and education level) or BMI indicate statistically significant differences (*p* < 0.05). NK: *Nutrition knowledge*; ATFL: *Attitude Toward Food Labels*; NR: *Reasons for Never Reading the Nutrition Information*; FCQ: *Food Choice Questionnaire*. * The first item of *Label-Reading Behavior*: occasional reading (options: never and rarely) and frequent reading (options: often and always).

**Table 5:** Mean values per cluster of the different items or constructs assessed in the questionnaire and label reading percentages for the participants who followed a diet.

	Mean Value			
	Cluster 1 (*n* = 86)	Cluster 2 (*n* = 90)	Cluster 3 (*n* = 70)	Cluster 4 (*n* = 52)
NK	10.8 ^b^	13.3 ^a^	12.9 ^a^	10.9 ^b^
ATFL	4.7	4.5	4.4	4.4
NR.1	2.6 ^c^	4.8 ^a^	4.0 ^b^	3.7 ^b^
NR.2	3.1 ^a^	1.8 ^b^	2.6 ^a^	2.9 ^a^
NR.3	3.3 ^a^	1.8 ^b^	2.1 ^b^	2.3 ^b^
NR.4	2.9 ^a^	1.8 ^c^	2.2 ^bc^	2.9 ^ab^
NR.5	3.2 ^a^	2.0 ^b^	2.9 ^a^	3.1 ^a^
NR.6	3.0 ^a^	2.3 ^b^	3.1 ^a^	3.6 ^a^
NR.7	3.2 ^a^	2.4 ^b^	3.4 ^a^	3.1 ^a^
NR.8	2.6 ^c^	2.2 ^c^	4.8 ^a^	3.3 ^b^
NR.9	2.7 ^b^	1.9 ^c^	2.3 ^bc^	4.6 ^a^
FCQ Health	5.8 ^b^	6.2 ^a^	6.2 ^a^	5.6 ^b^
FCQ Mood	5.6 ^ab^	6.0 ^a^	6.0 ^a^	5.4 ^b^
FCQ Easy	5.1	5.1	5.3	4.9
FCQ Proximity	5.5 ^bc^	5.9 ^ab^	6.0 ^a^	5.3 ^c^
FCQ Sensory appeal	5.7 ^b^	5.9 ^ab^	6.1 ^a^	5.5 ^b^
FCQ Natural content	5.4 ^ab^	5.8 ^a^	5.8 ^a^	5.0 ^b^
FCQ Cheap	4.0 ^ab^	3.5 ^b^	4.1 ^ab^	4.3 ^a^
FCQ Value for money	5.6 ^b^	5.8 ^ab^	6.1 ^a^	5.4 ^b^
FCQ Weight control	5.4 ^a^	5.4 ^a^	5.7 ^a^	4.8 ^b^
FCQ Familiarity	5.2	5.2	5.2	4.8
Fiber impact	20.9	17.6	18.2	17.9
Sugar impact	23.5 ^b^	26.8 ^a^	23.8 ^ab^	22.6 ^b^
Saturated fats impact	23.7	24.8	22.6	22.8
Salt impact	20.9	18.4	18.5	17.3
Mineral impact	18.6 ^a^	13.9 ^b^	14.9 ^b^	15.6 ^ab^
Carbohydrates impact	21.7	21.7	18.7	18.8
Cholesterol impact	22.5 ^a^	19.3 ^ab^	20.4 ^ab^	18.2 ^b^
Total fat impact	24.1	24.1	22.8	21.3
Protein impact	20.5	20.9	18.0	18.3
Caloric impact	23.6	24.7	23.6	21.8
Vitamin impact	19.2 ^a^	13.9 ^b^	15.9 ^ab^	15.6 ^ab^
* Occasional reading	4.7%	3.3%	2.9%	7.7%
* Frequent reading	68.6 ^ab^%	77.8 ^a^%	68.6 ^ab^%	53.8 ^b^%

^a–c^: Different letters in the same row and sociodemographic variables (sex, age group, and education level) or BMI indicate statistically significant differences (*p* < 0.05). NK: *Nutrition knowledge*; ATFL: *Attitude Toward Food Labels*; NR: *Reasons for Never Reading the Nutrition Information*; FCQ: *Food Choice Questionnaire*. * The first item of *Label-Reading Behavior*: occasional reading (options: never and rarely) and frequent reading (options: often and always).

**Table 6:** Mean values per cluster of the different items or constructs assessed in the questionnaire for the participants who did not follow a diet.

	Mean Value		
	Cluster 1 (*n* = 57)	Cluster 2 (*n* = 60)	Cluster 3 (*n* = 58)
NK	9.4 ^b^	11.1 ^ab^	12.1 ^a^
ATFL	4.3	4.3	4.5
NR.1	3.6 ^b^	4.3 ^ab^	4.9 ^a^
NR.2	3.2 ^ab^	3.7 ^a^	2.7 ^b^
NR.3	2.7	3.2	2.7
NR.4	3.9 ^a^	2.1 ^b^	2.5 ^b^
NR.5	3.8 ^a^	4.2 ^a^	2.4 ^b^
NR.6	4.2 ^a^	3.9 ^a^	2.3 ^b^
NR.7	3.6 ^b^	4.6 ^a^	2.8 ^c^
NR.8	3.4	3.4	3.4
NR.9	4.0 ^a^	2.6 ^b^	2.8 ^b^
FCQ Health	5.3 ^c^	5.8 ^b^	6.3 ^a^
FCQ Mood	5.3 ^c^	5.8 ^b^	6.3 ^a^
FCQ Easy	5.0	4.9	5.4
FCQ Proximity	5.1 ^b^	5.6 ^ab^	6.0 ^a^
FCQ Sensory appeal	5.5 ^b^	5.8 ^ab^	6.2 ^a^
FCQ Natural content	4.8 ^b^	5.4 ^a^	5.6 ^a^
FCQ Cheap	4.0	3.7	3.8
FCQ Value for money	5.3 ^b^	5.9 ^a^	6.0 ^a^
FCQ Weight control	4.5	4.9	5.0
FCQ Familiarity	4.8	5.1	5.1
Fiber impact	13.9	14.9	15.1
Sugar impact	17.2	19.8	20.2
Saturated fats impact	17.3 ^b^	21.3 ^ab^	21.5 ^a^
Salt impact	12.8	16.5	13.7
Mineral impact	12.2	12.8	11.8
Carbohydrates impact	14.4	16.8	18.4
Cholesterol impact	15.7	17.9	16.8
Total fat impact	17.7 ^b^	20.3 ^ab^	22.1 ^a^
Protein impact	13.7	16.3	16.3
Caloric impact	16.3 ^b^	20.5 ^ab^	21.2 ^a^
Vitamin impact	12.9	12.7	12.6
* Occasional reading	28.1%	13.3%	12.1%
* Frequent reading	35.1 ^b^%	60.0 ^a^%	75.9 ^a^%

^a–c^: Different letters in the same row and sociodemographic variables (sex, age group, and education level) or BMI indicate statistically significant differences (*p* < 0.05). NK: *Nutrition knowledge*; ATFL: *Attitude Toward Food Labels*; NR: *Reasons for Never Reading the Nutrition Information*; FCQ: *Food Choice Questionnaire*. * The first item of *Label-Reading Behavior*: occasional reading (options: never and rarely) and frequent reading (options: often and always).

## Data Availability

The data presented in this study are available on request from the corresponding author. Although consumer data have been anonymized, the data are not publicly available.

## Appendix A

**Table A1:** Dimensions, items, measurement scale, and source of the constructs assessed in the questionnaire.

Constructs	Items/Dimensions	Measurement Scale	Source
*Nutrition knowledge* (NK)	NK 1: *Lentils contain only few useful nutrients; therefore, their health benefit is not great*. NK 2: *If you have eaten high-fat foods, you can reverse the effects by eating apples*. NK 3: *If cream is whipped it contains less calories than in its liquid form*. NK 4: *A healthy meal should consist of half meat, a quarter vegetables, and a quarter side dishes*. NK 5: *Fat contains fewer calories than the same amount of fiber*. NK 6: *A salad dressing made with mayonnaise is as healthy as the same dressing made with mustard*. NK 7: *Fat is always bad for your health; you should therefore avoid it as much as possible*. NK 8: *Pasta with tomato sauce is healthier than pasta with mushroom and cream sauce*. NK 9: *A balanced diet implies eating all foods in the same amounts*. NK 10: *The health benefit of fruit and vegetables lies only in the supply of vitamins and minerals*. NK 11: *Bacon contains more calories than ham*. NK 12: *Oily fish (salmon, mackerel) contain healthier fats than red meat*. NK 13: *To eat healthily, you should eat less fat. Whether you also eat more fruit and vegetables does not matter*. NK 14: *A scoop of chocolate ice cream is just as healthy as a scoop of lemon sorbet*. NK 15: *The same amounts of beef steak and chicken breast contain an equal number of calories*. NK 16: *The same amounts of sugar and fat contain an equal number of calories*. NK 17: *A sandwich with mozzarella contains as many calories as the same sandwich with Gruyère cheese*. NK 18: *For healthy nutrition, dairy products should be consumed in the same amounts as fruit and vegetables*. NK 19: *Skimmed milk contains fewer minerals than full-fat milk*. NK 20: *Brown sugar is much healthier than white sugar*.	Three-point scale (true, false, and do not know).	[24]
*Attitude Toward Food Labels* (ATFL)	ATFL 1: *The Nutrition Facts label that appears on many food packages is a useful tool for consumers*. ATFL 2: The *nutrient information that is provided in the Nutrition Facts label is accurate*. ATFL 3: The *nutrition claims, such as “high fiber” and “no fat”, that appear on the front of food packages are truthful*. ATFL 4: The *health claims that appear on the front of food packages are truthful. An example of a health claim is: “Diet low in sodium may reduce the risk of high blood pressure, a disease associated with many factors”*.	Seven-point Likert scale (1 = strongly disagree to 7 = strongly agree). Extended from the original measurement scale.	[21]
*Reasons for Never Reading the* *Nutrition* *Information* (NR)	*I do not read the nutrition information because:* NR. 1: *I usually buy the same product; thus, I am familiar with the nutrition information*. NR. 2: *It takes too much time to read*. NR. 3: *I prefer getting information from other sources*. NR. 4: *I am just not interested*. NR. 5: *It is difficult to read*. NR. 6: *I really do not know what to do with the information*. NR. 7: *The information is not presented in the same way from one product to another*. NR. 8: *It is not always on products*. NR. 9: *I just pay attention to the price*.	Seven-point Likert scale (1 = strongly disagree to 7 = strongly agree). Modified from the original measurement scale.	[22]
*Food Choice* *Questionnaire* (FCQ)	*It is important to me that the food I eat on a typical day:* FCQ Health *Keeps me healthy* *Is nutritious* FCQ Mood *Helps me to cope with life* *Makes me feel good* FCQ Convenience *Is easy to prepare* *Can be bought in shops close to where I live or work* FCQ Sensory Appeal *Smells nice* *Tastes good* FCQ Natural Content *Contains no additives* *Contains natural ingredients* FCQ Price *Is cheap* *Is good value for money* FCQ Weight Control *Is low in calories* *Helps me control my weight* FCQ Familiarity *Is what I usually eat* *Is familiar*	Seven-point Likert scale (1 = strongly disagree to 7 = strongly agree). Modified scale according to Prescott et al. 2002.	[16]
*Perceived* *Importance of* *Nutrition Facts* (PI)	*Indicate the degree of importance of:* PI. 1: Dietary fiber PI. 2: Additional sugar PI. 3: Saturated fat PI. 4: Sodium PI. 5: Mineral PI. 6: Carbohydrate PI. 7: Cholesterol PI. 8: Total fat PI. 9: Protein PI. 10: Calories PI. 11: Vitamin	Seven-point scale (1 = not important o 7 = very important). Extended from the original measurement scale.	[23]
*Label-Reading* *Behavior* (LRB)	LRB. 1: *When you purchase a food product, do you look at the Nutrition Facts label on the package?* *I read the label to know the content of:* LRB. 2: Total fat LRB. 3: Calories LRB. 4: Saturated fat LRB. 5: Sodium LRB. 6: Cholesterol LRB.7: Sugar LRB. 8: Carbohydrate LRB. 9: Vitamin C LRB. 10: Calcium LRB. 11: Protein LRB. 12: Iron LRB. 13: Vitamin A LRB. 14: Dietary fiber	Five-point scale (1 = never to 5 = always).	[21]
